# OBML – Ontologies in Biomedicine and Life Sciences

**DOI:** 10.1186/2041-1480-2-S4-I1

**Published:** 2011-08-09

**Authors:** Heinrich Herre, Robert Hoehndorf, Janet Kelso, Frank Loebe, Stefan Schulz

**Affiliations:** 1 Institute for Medical Informatics, Statistics and Epidemiology (IMISE), University of Leipzig, Haertelstrasse 16-18, 04107 Leipzig, Germany; 2 Department of Genetics, Downing Street, CB2 3EH Cambridge, UK; 3 Department of Evolutionary Genetics, Max Planck Institute for Evolutionary Anthropology, Deutscher Platz 6, 04103 Leipzig, Germany; 4 Department of Computer Science, University of Leipzig, Johannisgasse 26, 04103 Leipzig, Germany; 5 Institute of Medical Informatics, Statistics and Documentation, Medical University of Graz, Auenbruggerplatz 2, 8036 Graz, Austria

## Abstract

The OBML 2010 workshop, held at the University of Mannheim on September 9-10, 2010, is the 2*^nd^* in a series of meetings organized by the Working Group “Ontologies in Biomedicine and Life Sciences” of the German Society of Computer Science (GI) and the German Society of Medical Informatics, Biometry and Epidemiology (GMDS). Integrating, processing and applying the rapidly expanding information generated in the life sciences — from public health to clinical care and molecular biology — is one of the most challenging problems that research in these fields is facing today. As the amounts of experimental data, clinical information and scientific knowledge increase, there is a growing need to promote interoperability of these resources, support formal analyses, and to pre-process knowledge for further use in problem solving and hypothesis formulation.

The OBML workshop series pursues the aim of gathering scientists who research topics related to life science ontologies, to exchange ideas, discuss new results and establish relationships. The OBML group promotes the collaboration between ontologists, computer scientists, bio-informaticians and applied logicians, as well as the cooperation with physicians, biologists, biochemists and biometricians, and supports the establishment of this new discipline in research and teaching. Research topics of OBML 2010 included medical informatics, Semantic Web applications, formal ontology, bio-ontologies, knowledge representation as well as the wide range of applications of biomedical ontologies to science and medicine. A total of 14 papers were presented, and from these we selected four manuscripts for inclusion in this special issue.

An interdisciplinary audience from all areas related to biomedical ontologies attended OBML 2010. In the future, OBML will continue as an annual meeting that aims to bridge the gap between theory and application of ontologies in the life sciences. The next event emphasizes the special topic of the *ontology of phenotypes*, in Berlin, Germany on October 6-7, 2011.

## Introduction

Utilizing the rapidly growing body of data and knowledge in the life sciences poses a severe challenge today. Integration of data and knowledge is particularly important in translating research outputs from molecular biology and genetics into improvements in the treatments of disease. A prime goal of genetics, molecular biology and biochemistry is to reveal the relation between molecular mechanisms and their resulting contribution to an organism’s phenotype. Recent advances in high-throughput sequencing technologies have facilitated the rapid identification of the genomes of a range of individual organisms. The development of high-throughput technologies in molecular biology has enabled the possibility for a paradigm shift towards the personalized treatment of disease based on an individual patient’s genetic markup. To facilitate this paradigm shift, biological knowledge about the disease mechanisms must be accessible to scientific and clinical analyses. In particular the molecular, genetic and phenotypic information that is relevant to understanding human disease can be investigated in organisms of different species that may serve as models for humans.

The challenge is to represent, compare and analyze information within and across domains and representation formats, and resulting from various analytical methods in order to produce knowledge bases that are amenable to scientific investigation and clinical application. Ontologies provide formal specifications and harmonized definitions of representational units (types, classes, concepts) and thus provide the building blocks for the computable modeling of domain knowledge. Methods in formal ontology support and enhance the semantic foundation, the standardization and the analysis of concepts related to a domain of reality and underpin the correct formalization of domain knowledge. Additionally, ontologies play an important role in the context of the Semantic Web promoting new paradigms of knowledge representation, processing and distribution.

Integration of data and knowledge in science benefits from a precise and formal characterization of data that is based on ontologies and the use of symbolic logic. In contrast to heuristic, approximate or statistical approaches, methods based on expressive logics provide a justification and explanation of the answers they provide and deliver a degree of certainty that is not achievable through other methods. They are therefore particularly suitable for the representation of scientific knowledge and can provide a layer based on which biomedical information can be verified, exchanged and integrated.

Ontologies also provide a rich graph-structure, which can be dynamically generated through automated inference, and which can be utilized to improve statistical methods or enable the use of distance metrics to measure semantic similarity. Ontologies based on expressive logics can further provide the means to verify results of scientific analyses, reject contradictory hypotheses and reveal the implications of scientific findings.

The OBML workshop series pursues the aim of gathering scientists who investigate topics related to life science ontologies, who exchange ideas, discuss new results and promote cooperations and collaborations. Research topics of OBML 2010 included medical informatics, Semantic Web applications, formal ontology, bio-ontologies, knowledge representation as well as the wide range of applications of biomedical ontologies to science and medicine. Out of this wide range of topics, we selected four manuscripts, which are contained in this special issue.

## Contributions to OBML 2010

### Extended articles in this issue

In the area of knowledge representation and applied ontology, the OBML workshop benefited from discussions of how to represent dispositions [1] and grains, components and mixtures [2] in biomedical ontologies. These topics are important for representing diseases, chemical entities and physiological processes that involve solutions such as blood. Dispositions are widely discussed both in philosophy [3-6], in the formal ontology community [7-10] as well as in the context of medical applications and representing disease [11,12]. A major challenge in representing dispositions is their context dependence, their dependence on a trigger, as well as the relation between dispositions and the processes that may be their realizations. Another problem arises due to the absence of relations with more than two arguments in description logics underlying the Web Ontology Language (OWL) [13,14]. The article “Representing dispositions” investigates how to represent dispositions in OWL, including their triggers and contexts, discusses applications in disease ontologies and ontologies of chemistry and provides an outlook on complex dispositions that can be realized through multiple kinds of processes or use multiple triggers. The paper “Grains, Components and Mixtures in Biomedical Ontologies” discusses competing representations of collectives (multiple entities of the same sort) for an adequate representation of substance mixtures. The authors argue that a strict dichotomy between (homogeneous) collectives and (heterogeneous) compounds, as advocated in previous work, is problematic. For instance, the distinction between isomeric subtypes of a molecule can be important in one use case but might be neglected in another one. Two different ways of representing mixtures are presented: (i) mixtures are the additions of two or more fractions of collectives, or (ii), mixtures are collectives of two or more types of granular parts. Using OWL-DL and the upper-level ontology BioTop [15], the authors demonstrate how the equivalence between both representations can be computed.

The article “Anatomy Ontologies and Potential Users: Bridging the Gap” evaluates the overlap of terms in anatomy ontologies and provides insight in how to align species-specific anatomies into a cross-species anatomy ontology. For this purpose, the authors evaluated how well the class names in anatomy ontologies fit the terms that are used by annotators. The authors selected three comprehensive data sets of annotations and evaluated the Foundational Model of Anatomy (FMA) [16] and the species-independent UBERON anatomy ontology [17] using lexical matching approaches. As a result, the authors identified a mismatch between the terms used by annotators and the terms provided by anatomy ontologies. These constitute short-comings in anatomy ontologies, which should be addressed by their developers to bridge the gap between the ontologies’ formal representation and their potential users.

Clinical applications of ontologies are the topic of the article titled “An Ontologically Founded Basic Architecture for Information Systems in Clinical and Epidemiological Research”. The authors present an architecture for clinical information systems founded in the General Formal Ontology (GFO) [18] and demonstrate applications to the representation of phenotypes as well as to clinical trials. Since the architecture is based on a top-level ontology, it is argued that such a meta-model can be applied in several scenarios, including clinical trial data and epidemiological research.

### Further contributions at the workshop

Several presentations covering a broad range of topics were given at the OBML 2010 workshop. The use of a semantic wiki, SBML2SMW, which is based on the Semantic MediaWiki architecture [19] and applied to the Systems Biology Markup Language [20] for application to the domain of systems biology was demonstrated [21]. SBML2SMW establishes a bi-directional information flow between a semantic wiki system and the domain of systems biology, and thereby enables the use of automated reasoning to discover hidden knowledge in biosimulation models.

Another demonstration of implementing ontology-based software in health care was given by C. Cocos and W. MacCaull [22], who illustrated how the Basic Formal Ontology [23] and its theory of roles can contribute to the implementation of access control policies in a healthcare information system.

Zaveri et al. [24] used the Linked OpenData cloud to evaluate the disparity between active areas of research and the global burden of disease. The presentation demonstrated a research program to automatically identify research areas and links between them, and illustrated potential visualization as well as analysis methods.

An important topic at OBML 2010 was knowledge representation, reasoning and foundations of biomedical ontologies, and several manuscripts contributed insights into these areas of knowledge. N. Grewe [25] provided a comprehensive discussion of strategies on representing *n*-ary relations in OWL. The topic of *n*-ary relations has applications from representing dispositions over the representation of processes to relations that contain temporal arguments.

The question whether anatomic cavities can be inflamed (e.g., by a verbatim interpretation of the term “Sinusitis”), or whether it must be the material boundary of a cavity such as a nasal sinus, was raised by J. Niggemann et al. [26], who illustrated the relevance of such questions for SNOMED CT concept definitions and the reliability of inferences.

Further manuscripts presented at the OBML workshop discussed the construction of an ontology for primary immuno-deficiencies [27], a cellular genealogical tree ontology [28], strategies for improving phenotype ontologies [29], the importance of simplifying ontologies to improve comprehensibility and understanding [30], as well as practical decisions on pre- vs. post-coordination in ontology engineering [31].

## Outlook

In 2010, the OBML workshop could gather an interdisciplinary audience from all areas related to biomedical ontologies. In the future, OBML will continue as a yearly event, organized by the working group, that aims to bridge the gap between theory and application of ontologies in the life sciences. To achieve this goal, the OBML working group will collaborate with the International Association for Ontology and its Applications (IAOA), the German Society of Computer Science (GI), and the German Society of Medical Informatics, Biometry and Epidemiology (GMDS). The OBML organizing and program committees will span all scientific disciplines associated with ontologies: from knowledge representation, logics and philosophy over Semantic Web technologies, natural language processing and statistics to applications in all systems, domains and levels of granularity studied in biomedicine.

The next OBML workshop will take place in Berlin on October 6-7, 2011, and it will be partly dedicated to the special topic of the *ontology of phenotypes*.

## Competing interests

The authors declare that they have no competing interests.
